# A Meta-Analysis of the Association between TNF-*α* **−**308G>A Polymorphism and Type 2 Diabetes Mellitus in Han Chinese Population

**DOI:** 10.1371/journal.pone.0059421

**Published:** 2013-03-19

**Authors:** Zheng-hui Liu, Yuan-lin Ding, Liang-chang Xiu, Hai-yan Pan, Yan Liang, Shou-qiang Zhong, Wei-wei Liu, Shao-qi Rao, Dan-li Kong

**Affiliations:** 1 Department of Epidemiology and Medical Statistics, School of Public Health, Guangdong Medical College, Dongguan, Guangdong, China; 2 Department of Endocrinology and Metabolism, Maoming People’s Hospital, Maoming, Guangdong, China; Harvard Medical School, United States of America

## Abstract

**Objective:**

A meta-analysis was applied to evaluate the associations between tumor necrosis factor-*α* (TNF-*α*) −308G>A (rs1800629) polymorphism and type 2 diabetes mellitus (T2DM).

**Methods:**

Hardy-Weinberg equilibrium (HWE) was employed to test genetic equilibrium among the genotypes of the selected literature. Power analysis was performed with the Power and Sample Size Calculation (PS) program. A fixed or random effect model was used on the basis of heterogeneity. Publication bias was quantified and examined with the Begg's funnel plot test and Egger's linear regression test. The meta-analysis was performed with Review Manager 5.1 and Stata 11.0.

**Results:**

There were 10 studies including 1425 T2DM patients and 1116 healthy control subjects involved in this meta-analysis. No significant publication bias was found in the studies. The pooled *ORs* (95% *CIs*) for TNF-*α* −308G>A of A vs. G allele and GA+AA vs. GG genotype were 1.63 (1.17–2.25) and 1.47 (1.17–1.85), respectively.

**Conclusion:**

This meta-analysis result suggested that TNF-*α* −308G>A polymorphism was strongly associated with T2DM risk, and A allele at this locus might be a susceptibility allele for the development of T2DM in Han Chinese population.

## Introduction

Diabetes mellitus (DM) has become a major health problem in the world. It is estimated that the population with DM will rise to 300 million in the year of 2025, and most of them are type 2 diabetes mellitus (T2DM) [1]. T2DM is a chronic complex disease with strong genetic components. It is well known that insulin resistance plays an important role in the development of T2DM. The pathogenesis of T2DM is generally considered being the results of interactions between multiple genetic susceptibility genes, and between genes and the environment [2]. Tumor necrosis factor-*α* (TNF-*α*) is a multifunctional cytokine produced by adipose tissue. It has been shown that TNF-*α* can affect insulin resistance by regulating adipocyte gene expression [3], [4], and that insulin resistance is an important pathophysiological mechanism of T2DM [5], [6].

**Figure 1:**
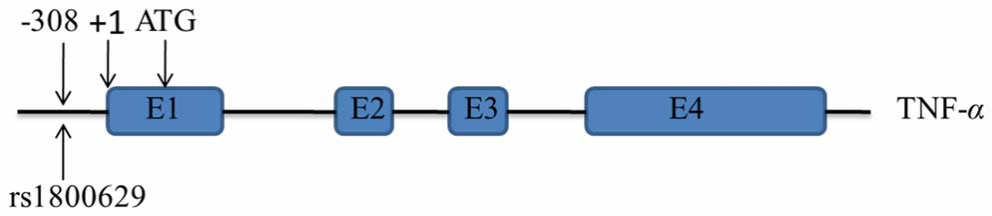
The schematic diagram of TNF-*α* −308G>A. In this graph, SNP rs1800629 locates on 5' upstream promoter region of TNF-*α* gene. “+1″ means the transcription initiation site, “ATG” represents the start codon, and “E1”, “E2”, “E3”, “E4” are 4 exons of TNF-*α* gene.

So far, a number of studies [7]–[12] examining the association between TNF-*α* polymorphism and T2DM risk have been published in different countries. However, the findings from these studies are largely inconsistent. Particularly in China, the obvious contradictory results across different studies were obtained, rendering interpretation of its genetic risk at large for Han Chinese people difficult and confusing. Most studies of Han Chinese population focused on the association of TNF-*α* −308G>A, a genetic variant in the promoter region of this gene, with T2DM risk, because it may play an important regulatory role for TNF-*α* production. The schematic diagram of TNF-α −308G>A together with the location of the candidate SNP rs1800629 was shown in Figure 1.

Nevertheless, the association between this polymorphism and T2DM susceptibility still remained unclear in Han Chinese population, with inconsistent findings across different studies. Since each single study was limited in providing sufficient information and adequate power for Han Chinese population, summarizing the results from these individual studies for obtaining a reliable and consolidated conclusion regarding TNF-*α* polymorphisms become essential. The aim of this meta-analysis was to integrate the findings from multiple studies to provide an overall assessment whether TNF-*α* −308G>A (rs1800629) polymorphism is associated with T2DM risk in a population of Han Chinese subjects.

## Materials and Methods

### Literature Databases

Major electronic literature databases were systematically searched, which included China National Knowledge Infrastructure (CNKI) database (http://www.cnki.net/), Chinese VIP database (http://www.cqvip.com/), Chinese Wanfang database (http://g.wanfangdata.com.cn/) and MEDLINE database (http://www.ncbi.nlm.nih.gov/pubmed), up to September 2012, for all publications about the association between TNF-*α* −308G>A polymorphism and T2DM in Han Chinese population. The keywords in this search were consisted of TNF-*α*, type 2 diabetes mellitus, polymorphism and Chinese/China.

### Selection Criteria

To be included in this meta-analysis, studies must meet the following criteria: case-control studies, available genotypic and allelic data or summarized frequencies, Chinese Han as the studied subjects, and the diagnosis of T2DM patients based on the 1999 WHO Diabetes Criteria (e.g., with fasting plasma glucose concentration of the subjects above 7.0 mmol/L were diagnosed as diabetes) [13]. If there were multiple publications from the same study group, the most recent study was included in this meta-analysis. However, if the study was a review, lecture, editorial or correspondence letter, it was excluded. Also, Hardy-Weinberg equilibrium (HWE) was checked for each study and the studies whose healthy control groups failed HWE were also excluded (to limit heterogeneity and ensure quality of data). Study quality was assessed using the guidelines for case-control studies [14]. Due to the criteria developed by Clark et al [15], a quality assessment score (as implemented in [16]–[18]) was estimated for the quality of each association study based on traditional epidemiological and genetic considerations. Using a 10-point scoring system, studies were scored as “good” if the score was 8 to 10, “moderate” if the score was 5 to 7 and “poor” if the score was less than 4 [19].

### Data Extraction

Two reviewers independently extracted data from relevant studies, including author, year of publication, geographic region, genotype distribution for T2DM patients and healthy control subjects, descriptive statistics on each study (e.g., gender, age, body mass index, total cholesterol, and triglyceride etc.) and *P*-values of HWE test in healthy control groups. Then, a group discussion was conducted to remedy any discrepancies in data collected by different reviewers.

### Statistical Analysis

This meta-analysis was performed with Review Manager 5.1 and Stata 11.0. Power analysis was performed using the Power and Sample Size Calculation (PS) program (http://biostat.mc.vanderbilt.edu/wiki/Main/PowerSampleSize) [20]. Heterogeneity among studies was examined with *χ* ^2^-based *Q* statistic [21]. If there was statistically significant heterogeneity among studies (*P*<0.05), a random effect model (Dersimonian-Laird method) was selected to merge data. Otherwise, a fixed effect model (Mantel-Haenszel method) was employed to analyze data. Odds ratios (*OR*s) with 95% confidence intervals (*CI*s) were calculated to assess the association between −308G>A of TNF-*α* and T2DM risk. Then, publication bias was estimated by Begg's funnel plot test and Egger's linear regression test. Finally, sensitivity analysis was performed to assess the influence of each individual study.

## Results

### Characteristics of Eligible Studies

At first, 11 studies of the association between TNF-*α* −308G>A (rs1800629) polymorphism and T2DM susceptibility in Han Chinese were identified. Then, one study [22] was excluded since it significantly deviated from HWE in healthy control group (*P*<0.05). Hence, there were actually 10 studies [23]–[32] to be used in this meta-analysis, with a total of 1425 patients of T2DM and 1116 healthy control subjects. The characteristics of the included studies were summarized in Table 1. Among these studies, eight were from Chinese Mainland, and the other two from Hong Kong. The mean age of all the subjects ranged from 36.1 to 60.5 years, and the gender was not evenly distributed in the 10 studies. Moreover, not all of the included studies were adjusted for covariates, such as body mass index, total cholesterol, triglyceride and so on. As a result, the information for these covariates was not shown in Table 1. The quality for these studies was rated to be moderate or above according to the quality assessment scores calculated.

**Table 1:** Characteristics of the eligible studies in this meta-analysis.

			T2DM/Control			
Author	Year	Region	Male (%)	Age (years)	n	*P*_HWE_
Chen J et al.	2009	Ningxia	52.0/56.4	56.9/52.9	100/101	0.5627
Ko GT et al.	2003	Hong Kong	27.4/34.7	38.2/36.8	339/202	0.2375
Lee SC et al.	2000	Hong Kong	38.9*	49.0*	238/121	0.3767
Liu HL et al.	2008	Hunan	53.9/55.7	48.2/46.5	245/122	0.5342
Pan HY et al.	2006	Heilongjiang	60.0/44.4	51.0/39.8	60/54	0.7213
Ren RZ et al.	2003	Fujian	60.0/51.7	58.6/57.7	70/60	0.5801
Sun B et al.	2006	Tianjin	52.0/50.0	60.5/60.4	100/50	0.4321
Wang LL et al.	2012	Jilin	58.0/45.1	52.9/40.6	100/113	0.3592
Wang ZJ et al.	2005	Hebei	51.7/52.6	57.0/55.0	118/78	0.5887
Zhang PA et al.	2003	Hubei	61.8/53.5	56.1/36.1	55/215	0.3071

* The combined data of T2DM and control.

*P*_HWE_, *P*-values of Hardy-Weinberg equilibrium test in healthy control groups.

### Power Analysis

Before this meta-analysis, power analysis was performed by using the PS program to prove whether the selected studies could offer adequate power. With data provided by the 10 studies, Mantel-Haenszel test, assuming that each 2×2 table consists of cases and controls selected from a different stratum that is defined by studies, could provide the power of 0.996 and 0.916 under the allelic and dominant genetic models, respectively.

### Association between TNF-*α* **−**308G>A and T2DM

**Figure 2:**
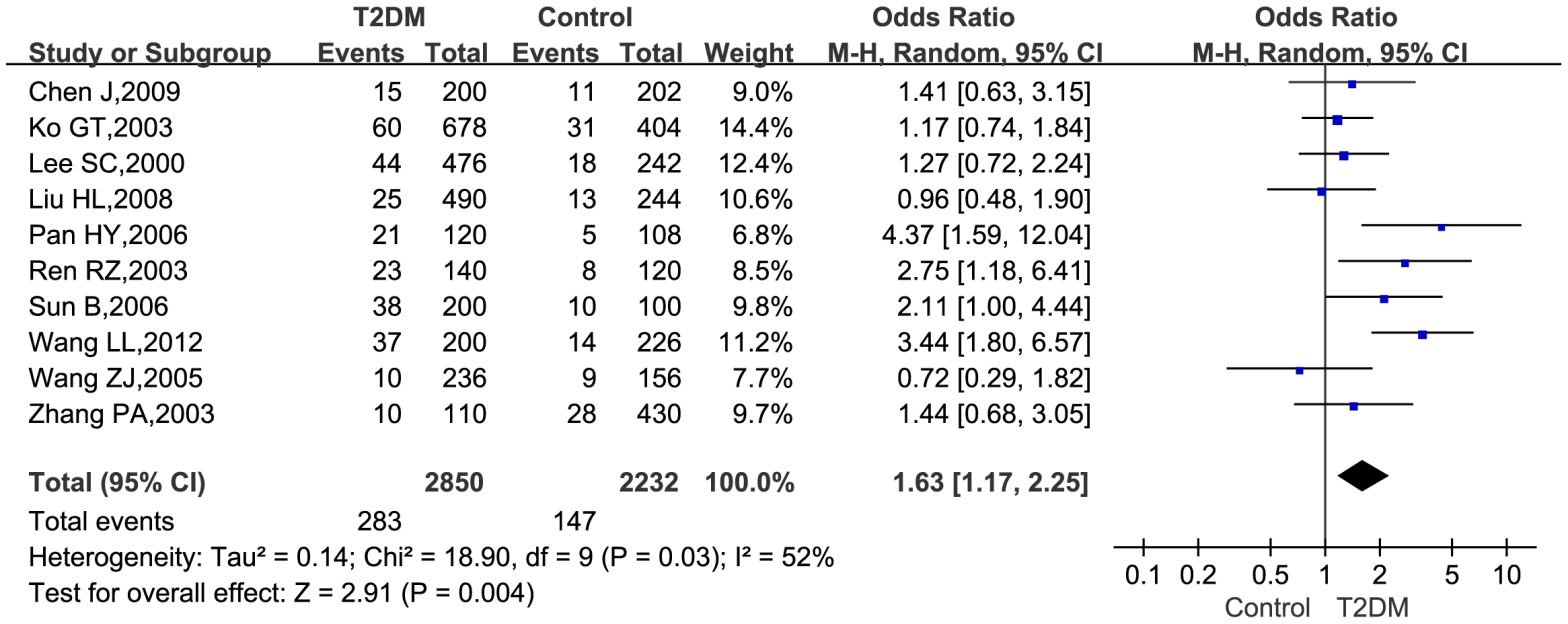
Forest plot of the association between T2DM and TNF-*α* −308G>A for A vs. G allele.

There was statistically significant allelic heterogeneity among the included studies (*P* = 0.03). Therefore, for estimating the overall allelic risk, a random effect model was selected to merge data. Figure 2 showed the *ORs* (95% *CIs*) estimate of TNF-*α* −308G>A (A vs. G allele) for each study and the overall estimate for the pooled data. The overall allelic *OR* (95% *CI*) was 1.63 (1.17–2.25), indicating that this allele brought a modest-size allelic risk of diabetes to Han Chinese population.

**Figure 3:**
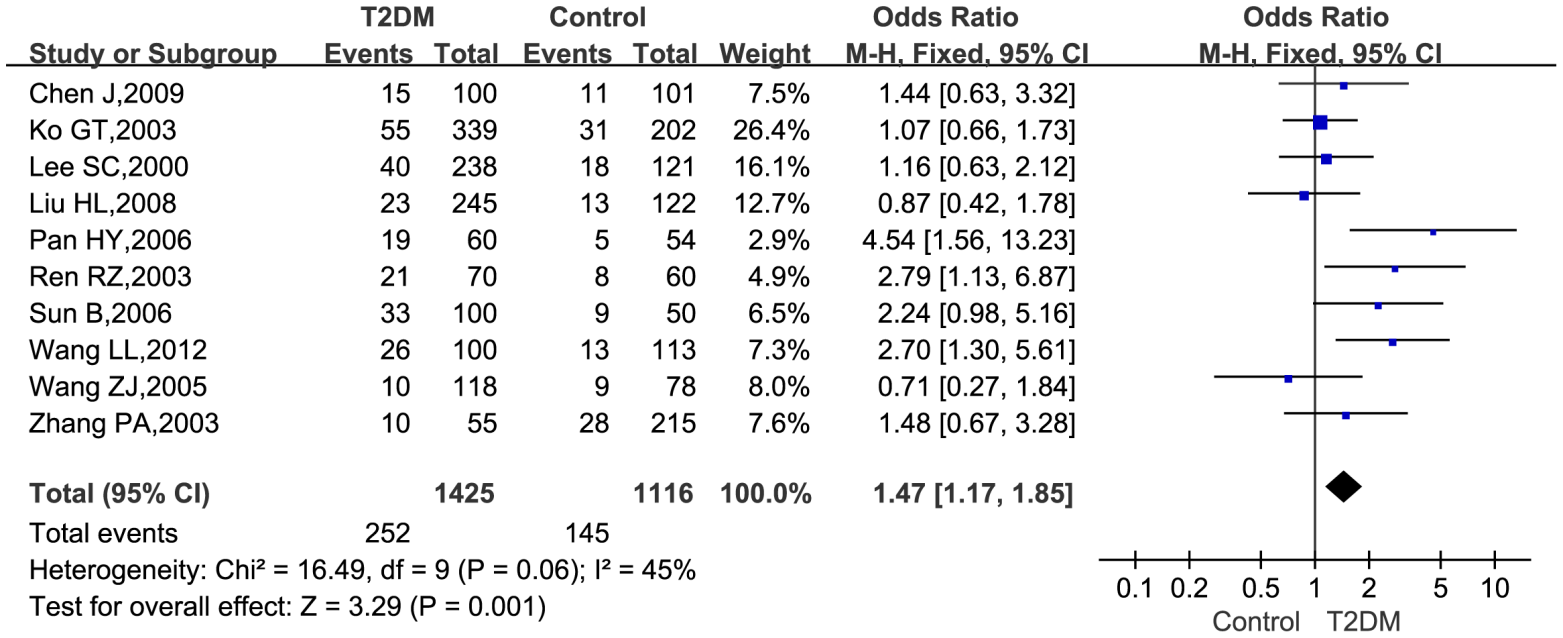
Forest plot of the association between T2DM and TNF-*α* −308G>A for GA+AA vs. GG genotype.

Association between genotypes (GA+AA vs. GG genotype) at TNF-*α* −308G>A locus and T2DM were further analyzed. Heterogeneity testing revealed that heterogeneity among the eligible studies was not statistically significant (*P* = 0.06). Hence, a fixed effect model was employed to analyze data. The pooled *OR* (95% *CI*) for TNF-*α* −308G>A under the dominant model (GA+AA vs. GG genotype) was 1.47 (1.17–1.85) (Figure 3), also interpreted to be a modest-size risk of diabetes.

### Publication Bias

**Figure 4:**
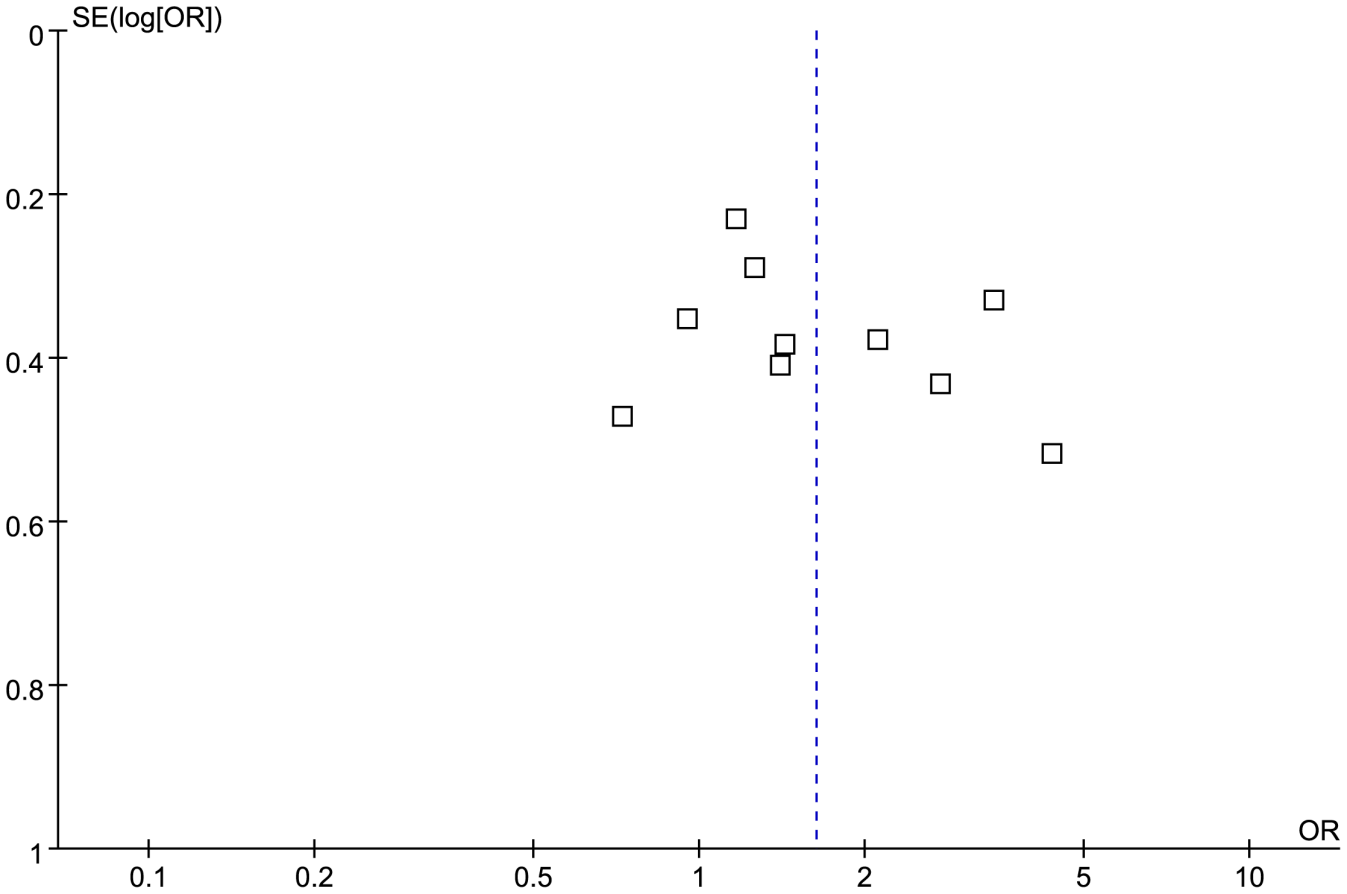
Funnel plot of the association between T2DM and TNF-*α* −308G>A for A vs. G allele.

**Figure 5:**
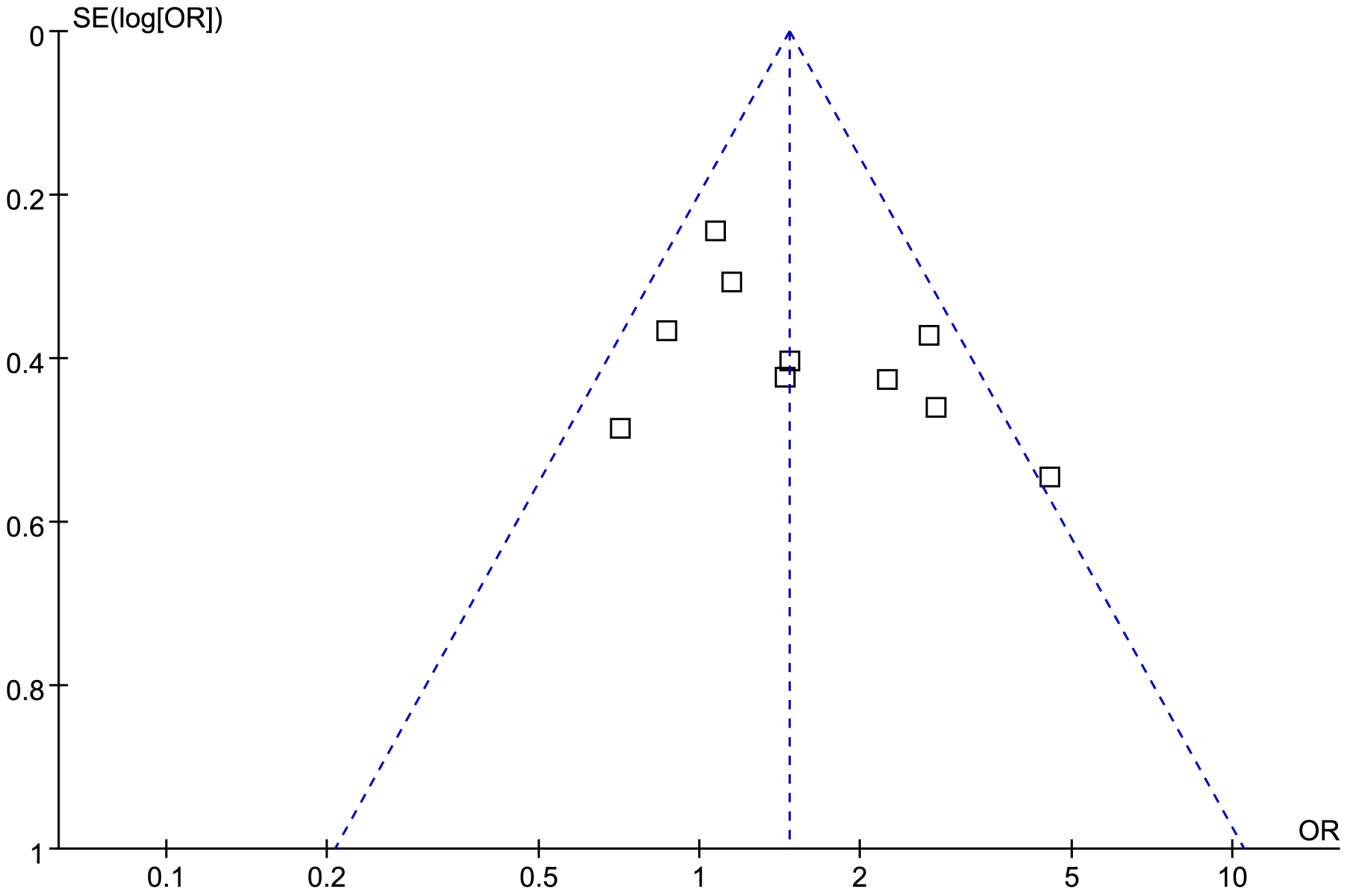
Funnel plot of the association between T2DM and TNF-*α* −308G>A for GA+AA vs. GG genotype.

Begg's funnel plot test and Egger's linear regression test were performed to assess any publication bias. The funnel plots of the association between T2DM and TNF-*α* −308G>A for A vs. G allele and GA+AA vs. GG genotype were shown in Figures 4 and 5, respectively. The *P*-values for Begg's test of TNF-*α* −308G>A A vs. G allele and GA+AA vs. GG genotype were 0.531 and 0.180, respectively, and the *P*-values for Egger's linear regression test were 0.323 and 0.123, respectively (Table 2). The shapes of the two funnel plots were symmetrical at large, and there were no statistical significance based on the *P*-values of Begg's test and Egger's linear regression test, suggesting no evidence of publication bias among the included studies.

**Table 2:** Begg's and Egger's test of publication bias for the association between TNF-*α*-308G>A and T2DM.

	Begg's test		Egger's test	
Comparison	*z*	*P*	*t*	*P*
A vs. G	0.63	0.531	1.05	0.323
GA+AA vs. GG	1.34	0.180	1.73	0.123

### Sensitivity Analysis

Finally, a sensitivity analysis was performed to evaluate the influence of individual studies. A leave-one-out procedure (i.e., omitting one study at a time) was used to estimate the contribution of a single study to the pooled *OR* value. This sensitivity analysis demonstrated that none of the studies influenced the pooled *OR* greatly. The leave-one-out *ORs* estimate ranged from 1.46 (1.08–1.96) to 1.72 (1.19–2.48) for A vs. G allele, and 1.37 (1.08–1.75) to 1.61 (1.24–2.10) for GA+AA vs. GG genotype, respectively, suggesting that the pooled estimates of allelic and genotypic risks obtained in this study were both stable and reliable.

## Discussion

TNF-*α* gene, situated on human chromosome 6p21.3, encodes a multifunctional proinflammatory cytokine, which plays the most important role in the development of T2DM. It has been well documented that high expression of TNF-*α* in adipose tissue is related to insulin resistance, considered to be an important pathogenic mechanism for the development of T2DM [33].

Recently, a number of candidate-gene studies for diabetes in Han Chinese population have been reported, and the association between TNF-*α* −308G>A (rs1800629) and T2DM is a hot focus. By searching literature, we found that the findings regarding this association were of obvious discrepancies across different studies. Hence, it is highly demanded to systematically assess the association of TNF-*α* −308G>A with T2DM prior to translation of this genetic finding into clinical practice.

To the best of our knowledge, this study was the first meta-analysis to comprehensively examine the association between TNF-*α* −308G>A and T2DM in Han Chinese population, and the pooled data from 10 eligible studies contained 1425 T2DM patients and 1116 healthy subjects. This meta-analysis showed that there was statistically significant association between TNF-*α* −308G>A polymorphism and T2DM risk in Han Chinese population, demonstrating that TNF-*α* −308G>A was an important risk factor for T2DM in Han Chinese population. This conclusion is largely consistent with a previous meta-analysis examining this association in Asian populations [34]. However, the worldwide meta-analysis of the studies of very different ethnical populations (divided into Asian, European and others), performed by Feng RN et al. [35], indicated that TNF-*α* −308G>A gene polymorphism was not associated with T2DM risk in these large racial divisions. These discrepancies suggest that the genetic effect of TNF-*α* −308G>A is race-specific. While it imposes a significant risk on Han Chinese population, TNF-*α* −308G>A polymorphism might not be associated with T2DM risk in other racial populations such as Caucasians [7], [12], [36], [37], Japanese [38], [39] and Indians [40]. In addition to ethnic differences, a list of epidemiological and design factors (e.g., gender, age, life style, study design or limited sample size) may explain the contradictory results obtained in different studies as well.

To avoid potential racial admixture, we restricted our analysis to Han Chinese in this study. All of the 10 included studies were of Han Chinese ancestry. Nevertheless, for Han Chinese population who live in a vast country, geographical and culture differences might be additional factors causing genetic heterogeneity. In this meta-analysis, five of the included studies were from northern China, and the remaining five from southern China. The culture and habits, such as personality, diet, living environment, customs and so on, in these two large geographic areas, were very different, and the genetic communications between these two geographic areas might not be as frequently as within each geographic area, which may lead to population stratification. The fact that significant between-study heterogeneity in allelic association and marginal significance in genotypic association across the 10 studies might support our perspective. Other factors (e.g., age, sex, sample size, genotyping method, gene-gene and gene-environment interactions) might contribute to heterogeneity as well. Unfortunately, due to limited number of studies and lack of size of samples, we did not perform any stratified analysis regarding these potential confounding factors.

Finally, we should recognize several limitations of this study. First, we did not take into account the impact of several covariates (the potential confounding factors) on the results of this meta-analysis due to incomplete data for several studies. Second, because of lack of the data for individuals’ environmental exposures, we did not further explore the interactions of TNF-*α* gene with various environmental factors. Finally, only single SNP (rs1800629) in TNF-*α* gene was analyzed in this study, and whether additional genetic variants of either functional or polymorphic contribute to this gene remains unclear, which may lead to underestimation of its overall genetic effect on T2DM susceptibility. Thus, constructing its spectrum of genetic variants is required for comprehensive assessment of its role in conferring susceptibility to T2DM.

In conclusion, by systematically integrating 10 well-characterized association studies for Han Chinese, with 1425 T2DM patients and 1116 healthy subjects, we found that TNF-*α* −308G>A (rs1800629) polymorphism were statistically significantly associated with T2DM in Han Chinese population, suggesting that A allele at this locus increases the risk for the development of T2DM in this population. However, in our view, more large-scale independent studies or larger sample sizes are still needed to consolidate this finding in the future. Furthermore, for a complex disease like T2DM, which is considered being the result of the sophisticated interplays between multiple genes, and their interactions with environment, a rational and extended research is to further identify the genes and environmental triggers interacting with TNF-*α* −308G>A and the functional involvement of this polymorphism leading to the molecular pathogenesis of T2DM.

## Supporting Information

Figure S1 **PRISMA flow diagram of including studies.**
(DOC)

Checklist S1 **PRISMA checklist of the association between TNF-** ***α*** −**308G>A and T2DM.**
(DOC)
