# Supplementary material for: A Meta-Analysis of the Association between TNF-α −308G>A Polymorphism and Type 2 Diabetes Mellitus in Han Chinese Population
Source: PLoS One. 2013 Mar 19;8(3):e59421. doi: 10.1371/journal.pone.0059421 (PMC3601959; doi:10.1371/journal.pone.0059421)
Supplement: Figure S1 — PRISMA flow diagram of including studies. (DOC) [file pone.0059421.s001.doc]

**Figure S1. The Flow Diagram of Including Studies.**

Potentially relevant studies identified and screened for retrieval (n=2549)

Studies excluded, without polymorphism reported (n=2397)

Studies retrieved for more detailed evaluation (n=152)

Potentially appropriate studies to be included in the meta-analysis (n=17)

Studies included in this meta-analysis (n=10)

Studies excluded, with not Han Chinese or China (n=135)

Studies excluded, with duplicate published or deviated from HWE in the healthy control group (n=7)
