# Supplementary material for: A Meta-Analysis of the Association between TNF-α −308G>A Polymorphism and Type 2 Diabetes Mellitus in Han Chinese Population
Source: PLoS One. 2013 Mar 19;8(3):e59421. doi: 10.1371/journal.pone.0059421 (PMC3601959; doi:10.1371/journal.pone.0059421)
Supplement: Checklist S1 — PRISMA checklist of the association between TNF- α −308G>A and T2DM. (DOC) [file pone.0059421.s002.doc]

**Checklist S1.** PRISMA checklist of the association between TNF-*α* -308G>A and T2DM.

| **Section/topic** | **Checklist item** |
| --- | --- |
| **Title** | |
| Title | A meta-analysis of the association between TNF-*α* -308G>A polymorphism and type 2 diabetes mellitus in Han Chinese population |
| **Abstract** | |
| Structured summary | A meta-analysis was applied to evaluate the associations between tumor necrosis factor-α (TNF-α) -308G>A (rs1800629) polymorphism and type 2 diabetes mellitus (T2DM). Major electronic literature databases were systematically searched, which included China National Knowledge Infrastructure (CNKI) database (http://www.cnki.net/), Chinese VIP database (http://www.cqvip.com/), Chinese Wanfang database (http://g.wanfangdata.com.cn/) and MEDLINE database (http://www.ncbi.nlm.nih.gov/pubmed). To be included in this meta-analysis, studies must meet the following criteria: case-control studies, available genotypic and allelic data or summarized frequencies, Chinese Han as the studied subjects, and the diagnosis of T2DM patients based on the 1999 WHO Diabetes Criteria. There were 10 studies including 1425 T2DM patients and 1116 healthy control subjects involved in this meta-analysis. No significant publication bias was found in the studies. The pooled ORs (95% CIs) for TNF-α -308G>A of A vs. G allele and GA+AA vs. GG genotype were 1.63 (1.17-2.25) and 1.47 (1.17-1.85), respectively. This meta-analysis results suggested that TNF-α -308G>A polymorphism was strongly associated with T2DM risk, and A allele at this locus might be a susceptibility allele for the development of T2DM in Han Chinese population. |
| **Introduction** | |
| Rationale | Tumor necrosis factor-α (TNF-α) is a multifunctional cytokine produced by adipose tissue. It has been shown that TNF-α can affect insulin resistance by regulating adipocyte gene expression, and that insulin resistance is an important pathophysiological mechanism of T2DM. |
| Objectives | The aim of this meta-analysis is to integrate the findings from multiple studies to determine whether TNF-*α* -308G>A polymorphism is associated T2DM risk in Han Chinese population. |
| Type of study design | Case-control study |
| Hypothesis | TNF-*α* -308G>A polymorphism may increase the risk of T2DM. |
| Study population | Han Chinese population |
| Compare objects | T2DM patients and healthy control subjects,  A vs. G allele and GA+AA vs. GG genotype of TNF-*α* -308G>A |
| **Methods** | |
| Search strategy | We searched major electronic literature databases which include CNKI database, Chinese VIP database, Chinese Wanfang database and MEDLINE database, up to September 2012 for all publications. The keywords included TNF-*α*, T2DM, polymorphism and Chinese/China. |
| Selection criteria | Case-control study, available genotype and allele data or summarized frequencies, Chinese Han as the studied subjects, obeying HWE, no multiple publications, and the diagnosis of T2DM based on the 1999 WHO Diabetes Criteria. |
| Quality assessment | Blinded screening and a quality assessment score was developed to evaluate the quality of included studies. |
| Data extraction | Two reviewers independently extracted data from relevant studies, including author, year of publication, region, genotype distribution among T2DM group and healthy control subjects and *P* value for HWE test in healthy control group. |
| Statistical analysis software | Manager 5.1 and Stata 11.0 |
| Test of heterogeneity | *χ*^2^-based *Q* statistic |
| Data consolidation | A fixed or random effect model was used. |
| Risk of bias across studies | Publication bias was estimated by Begg's test and Egger's linear regression test, selective reporting within studies also existed. |
| Additional analysis | Sensitivity analysis was performed to assess the influence of each individual study. |
| Summary measures | Odds ratios, 95% confidence intervals |
| **Results** | |
| Study selection | There were actually 10 studies to be used in the meta-analysis, with a total of 1425 T2DM patients and 1116 healthy control subjects. Figure S1 showed the flow diagram of including studies. |
| Study characteristics | Table 1 |
| Results of meta-analysis | Figures 2 and 3 |
| Results of publication bias | Figures 4 and 5, Table 2 |
| Results of sensitivity analysis | None of the individual study influenced the pooled *OR*, the results of our study were both stable and reliable. |
| **Discussion** | |
| Heterogeneity | Geographical and culture differences and other factors might contribute to heterogeneity. |
| Limitations | First, we did not take into account the impact of several covariates (the potential confounding factors) on the results of this meta-analysis due to incomplete data for several studies. Second, because of lack of the data for individuals’ environmental exposures, we did not further explore the interactions of TNF-α gene with various environmental factors. Finally, only single SNP (rs1800629) in TNF-α gene was analyzed in this study, and whether additional genetic variants of either functional or polymorphic contribute to this gene remains unclear, which may lead to underestimation of its overall genetic effect on T2DM susceptibility. Thus, constructing its spectrum of genetic variants is required for comprehensive assessment of its role in conferring susceptibility to T2DM. |
| Conclusions | TNF-*α* -308G>A polymorphism are strongly associated with T2DM, and TNF-*α* -308G>A A allele might be a susceptibility allele for T2DM in Han Chinese population. |
| Implications for future research | More large-scale independent studies are still needed to consolidate this finding in the future. Furthermore, for a complex disease like T2DM, which is considered being the result of the sophisticated interplays between multiple genes, and their interactions with environment, a rational extended research is to further identify the genes and environmental triggers interacting with TNF-α -308G>A and the functional involvement of this polymorphism leading to the molecular pathogenesis of T2DM. |
| **Funding** | |
| Funding | This study was supported by the National Natural Science Foundation of China (grant nos. 81273166, 30830104 and 31071166), National Natural Science Foundation of Guangdong Province (grant no. S2012010008271), Science and Technology Planning Project of Guangdong Province (grant no. 2009A030301004), Dongguan City Science and Technology Project (grant nos. 2012105102010, 2012108102058 and 2011108101015,) and the funds from Guangdong Medical College (grant nos. STIF201121 STIF201122, XG1001 and XZ1105). The funders had no role in study design, data collection and analysis, decision to publish, or preparation of the manuscript. |
